# COGNITIVE FUNCTION, ACTIVITY MEANINGFULNESS, AND SOCIAL ENGAGEMENT AMONG OLDER ADULTS WITH DEMENTIA

**DOI:** 10.1093/geroni/igad104.0186

**Published:** 2023-12-21

**Authors:** Shinyi Wu, Yi-Hsuan Tung, Iris Chi

**Affiliations:** University of Southern California, Los Angeles, California, United States; University of Southern California, Los Angeles, California, United States; USC, Los Angeles, California, United States

## Abstract

Hess’s selective engagement theory (SET) suggests that individuals facing aging-related decline tend to selectively allocate cognitive resources in activity engagement. This selectivity can be mediated or moderated by motivational factors (e.g., activity meaningfulness). Yet, empirical research on SET and older adults with dementia is limited. This study aims to examine how cognitive function and activity meaningfulness affect social engagement among this population. Data is from the 2016 National Health and Aging Trends Study (N= 1,585 participants aged 65+ with dementia). Engagement in four social activities including visiting family or friends (VF), attending religious activities (RA), participating in group activities (GA), and going out for enjoyment (OE) were analyzed in separate multivariate logistic regression models with cognitive function, the corresponding meaningfulness (a mediator and a moderator), and covariates (age, gender, race, living alone, self-rated health, ADL, IADL, and depressive symptoms). Results showed that, of the four social activities, cognitive function was only significantly associated with RA and GA engagement. RA meaningfulness moderates the association between cognitive function and RA engagement, in that high RA meaningfulness shows high RA engagement, while low RA meaningfulness shows low RA engagement and in a negative association with cognitive function. GA meaningfulness partially mediates the relationship between cognitive function and GA engagement. These findings support SET by showing cognitive resources-related selection effects on activity engagement and the roles of activity meaningfulness in selectivity effects. The implication highlights the importance of sustaining both cognitive function and motivational factors to promote social engagement among older adults with dementia.

